# Authors response on Schick et al. 2017 “An experiment of the impact of a neonicotinoid pesticide on honey bees; the value of a formal analysis of the data”. Environ Sci Eur (2017)

**DOI:** 10.1186/s12302-016-0102-9

**Published:** 2017-01-23

**Authors:** Peter Campbell, Mike Coulson, Keith Ward

**Affiliations:** Jealotts Hill International Research Centre, Syngenta, Bracknell, Berkshire RG42 6EY UK

**Keywords:** Thiamethoxam, Honeybee, Field studies, Statistical analysis, Critical review

## Abstract

Whilst a formal statistical analysis of any experimental data is always preferable in principle, in the case of Pilling et al. (PLoS ONE 8:e77193, 2013), it is hard to see how the results of any formal analysis—including those provided by Schick et al.—could be considered reliable. Regardless of the issue of statistical analysis, there was a wealth of valuable and novel biological and chemical residue data generated under field conditions of use in Pilling et al., which when taken into consideration alongside other relevant available published data and information (i.e. expert judgement) demonstrated a low risk to honeybees from thiamethoxam when used as a seed treatment on oilseed rape. Indeed, similar conclusions have been reported in subsequent published honeybee field studies using thiamethoxam seed-treated oilseed rape, thus supporting the original conclusions of Pilling et al.

## Background

The focus of Schick et al.’s 2017 [1] paper is field trials initiated over 10 years ago (2005–2008) by Syngenta in collaboration with Eurofins Contract Research Organisation. At the time of conduct, these field trials were the most extensive regulatory field testing trials ever undertaken to investigate the safety of neonicotinoids to honeybees. The field testing programme included 12 separate pollen and nectar field residue trials and five long-term (over 4 consecutive years) field effect trials on honeybees carried out in four geographically widespread locations in France. These trials were carried out for product registration purposes under the regulatory requirements of the European Plant Protection Product Directive 91/414/EEC. Subsequently, in 2013, in response to a call for industry to be more transparent with regard to its neonicotinoid honeybee data, Syngenta took the decision to publish the data from these studies [2]. In the summer of 2014, the authors of Pilling et al. [2] were approached by Schick et al., who requested to have access to the raw data behind the Pilling et al.’s [2] paper, in order to carry out their own statistical analysis of the data. In the spirit of openness and cooperation, the data were provided as requested.

## Methodology followed by Pilling et al. 2013

Schick et al. [1] criticise the methodology followed by Pilling et al. [2], in particular with regard to the lack of formal statistical analysis of the data. However, the field trials published in Pilling et al. [2] followed the only internationally agreed field test guidelines for honeybees with pesticides that were available at that time, i.e. EPPO 170 [3]. This EPPO 170 guideline was developed by the leading experts in the area of pesticide testing of honeybees at that time. The reason for a lack of formal statistical analysis of the data was clearly explained both in the original Pilling et al.’s paper [2], a published response of the authors of Pilling et al. [4] to a critical review [5] of Pilling et al. [2], as well as in a published revision of EPPO 170 Honeybee Field Testing Guideline [6]. All clearly state that although adequate statistical replication of field plots is desirable, it is not practically feasible because of the isolation requirements of the study design, i.e. replicate plots should be minimum of 2 ha in size and there should be 2–3 km between treatment and control plots and 2–3 km between treated/control plots and alternative forage in the landscape. Schick et al. [1] criticise the use of “expert judgement” in Pilling et al. [2]. However, in EPPO 170 honeybee field test guideline [6] it clearly states that the use of expert judgement is specifically required in order “to assess the biological significance of any effects seen in the context of each colony and the test conditions”.

## Statistical approach of Schick et al.

In their analysis, Schick et al. categorise the mortality data into ‘before’, ‘during’ and ‘after’ exposure. However, the Pilling et al.’s [2] trials were not designed as 4 repeated independent annual studies as is suggested by this categorisation and analysis. That is, this was a 4-year experiment investigating potential accumulative effects from 4 annual repeated exposures. Therefore, the only true ‘before’ period is the data reported from first measurements taken prior to exposure to the flowering crop in year 1. All other subsequent hive measurement could have been affected by that first exposure in year 1, since hive products are stored and fed to bees over a longer period.

With regard to the data analysis carried out by Schick et al., they are in danger of confusing two separate issues. The first is whether an approach based on estimated effect sizes and 95% confidence intervals is more informative than the one based solely on tests of significance. The second is whether either approach can generate reliable results given the limitations of the experimental design. Regardless of which approach is chosen, there is only ever one degree of freedom (*df*) for experimental error for oilseed rape and only ever two *df* for experimental error for maize. In both cases, it can be argued that this number of *df* is simply too few to generate a reliable estimate of experimental error. Also, with so few data points in any given analysis (i.e. four for oilseed rape and six for maize), it is difficult to see how it can be verified that the data meet the underlying assumptions upon which the validity of the analyses depend. Moreover, with so few data points it is difficult to see how Schick et al. could justify their choice of error distribution and link function ahead of other viable alternatives; this is important because alternative but equally justifiable choices could lead to very different results, particularly with regard to the whereabouts of the upper confidence limits. Thus, the Schick et al.’s [1] analyses—or indeed any other set of formal analyses—cannot be considered to give reliable results in this instance. Therefore, the original decision of Pilling et al. [2] to not subject the data in question to formal statistical analysis would appear entirely reasonable but of course others may take a different view. It should also be borne in mind that the Schick et al.’s [1] results are entirely dependent on their selected approach to condense a very large, complicated and multi-dimensional dataset into a handful of numbers (four for oilseed rape, six for maize) per endpoint, which could then be subjected to statistical analysis. There are clearly many ways in which this could have been done, and as has already been pointed out, the division of time points into “Before”, “During” and “After” is not one that is appropriate from a biological perspective.

Ecotoxicological field testing and risk assessment for pesticides is a continually evolving area, as shown by the regular review of guidelines for regulatory testing. It is for this reason that Syngenta co-sponsored with Bayer a bespoke landscape-level honeybee neonicotinoid field effect study with UK Centre for Ecology and Hydrology [7]. This bespoke study carried out at an unprecedented scale (i.e. multiple sites across, UK, Germany and Hungary) had sufficient statistical replication to allow a more meaningful formal analysis of the data and it is expected that the results of this study will be published in early 2017. The size of this study was such that had Syngenta and Bayer not sponsored it, the resources required would have virtually excluded it from being conducted.

## Robustness of the low-risk conclusion for thiamethoxam to bees

Schick et al. [1] criticise the conclusion of low risk to honeybees in Pilling et al. [2] based on the lack of statistical power of the study. However, whilst the lack of statistical power was recognised in the original paper [2], it should be noted that the conclusion of low risk was based on an expert analysis of the wealth of biological and chemical residue data generated from this and other published and unpublished studies. This included a comparison of the field reported pollen and nectar residue data with laboratory acute and chronic toxicity data for thiamethoxam. Residues of thiamethoxam in field collected pollen and nectar reported in Pilling et al. [2] were <1.0 ng/g for pollen and <0.5 ng/g for nectar. This is well below bee toxicity threshold values for thiamethoxam in worst case laboratory toxicity studies [8]. Field residues of this scale would be 0.3–1.4% of the acute oral dose based on a bee consuming 128 mg nectar per day [9]. Using a similar approach, no mortality would be expected after a chronic exposure even if a bee consumes 10 times its body weight in nectar per day. Therefore, no adult mortality effects would have been expected in this study.

In addition, since the publication of Pilling et al. [2], there have been two further published field studies which confirm the low-risk conclusions of Pilling et al. [2]. Thompson et al. [10] reported no sub-lethal effects of thiamethoxam on honeybee foraging behaviour using radio frequency identification tracking technology and Henry et al. [11] reported no colony-level effects of thiamethoxam on honeybees in their landscape analysis. The latter reported that even where increased forager loss is observed, colonies compensate for the excess mortality so as to preserve unaltered performance in terms of population size and honey production, underlining the importance of biological significance in defining risk.

## Conclusion

Schick et al. [1] focus on the argument that due to the low statistical power of the study design, this study cannot be used to rule out the possibility of an adverse effect. However, they fail to acknowledge the wealth of informative data generated from these field trials that equally provides no evidence for an adverse effect either. Pilling et al.’s [2] paper was extensively reviewed by five referees during the original review process, followed by a 2nd post-publication independent editorial review, where it was concluded that this paper was a useful addition to scientific literature. In the end, the conclusion of low risk in Pilling et al. [2] was informed by an expert analysis of the full biological and chemical data generated in this study as well as the supporting literature quoted at the time. Importantly, it should be noted that subsequent published honeybee field effect studies conducted with thiamethoxam seed-treated oilseed rape have reported similar conclusions to Pilling et al. [2], i.e. a low risk to honeybees under field conditions of use.
